# Are 50 years enough to prove the brilliant Dilman’s idea?

**DOI:** 10.18632/oncotarget.28097

**Published:** 2022-01-10

**Authors:** Vladimir N. Anisimov

**Keywords:** antidiabetic biguanides, metformin, cancer prevention, milestones

Fifty years ago *The Lancet* published a paper by Dilman in which for the first time it was suggested that antidiabetic biguanides may be promising potent anti-cancer and anti-aging drugs [1]. In the mid-70s of 20th century its author initiated a series of experiments in mice and rats aimed to prove this suggestion at the N.N. Petrov Research Institute of Oncology (Leningrad, USSR). In 1974, he showed for the first time that 1-phenylethylbiguanide (phenformin, PF) suppressed mammary carcinogenesis induced by 7,12-dimethylbenz(a)anthracene in female rats [2]. Five years later, the first report on the inhibitory effect of PF on spontaneous mammary carcinogenesis, and life extension in female C3H/Sn was published [3]. At the same time, it was reported that 1-butylbiguanide (buformin, BF) and PF slowed the aging of the reproductive system, prolonged the lifespan and inhibited spontaneous tumor development in female rats. Then, several papers reported data on the capacity of antidiabetic biguanides to prevent chemically- and radiation-induced carcinogenesis in the colon, kidney, nervous system, lymphoid and soft tissues in rodents [4]. During the next 20 years, only one article on the inhibitory effect of metformin on carcinogenesis in the pancreas of hamsters was published. In 2005, it was first shown that metformin prolonged the lifespan and inhibited mammary carcinogenesis in female transgenic HER-2/neu mice [5] and reduced the risk of breast cancer in women [6]. These articles lead to a surge in publications on metformin and cancer. According to PubMed, in 1971 only Dilman’s paper [1] published in *The Lancet* was dedicated to biguanides and cancer. However, in 2005 the number of articles on metformin and cancer was 95, in 2010–175 and in 2020–696, and a total 5750 articles of the years. Then, for the first time it was shown that metformin was not an effective anti-carcinogen in adult (9 months) and old (15 months) female SHR mice [7] and in 2-year-old male C57BL/6J mice [8]. Since 1974 till present, the anti-carcinogenic effect of antidiabetic biguanides has been studied, using 21 strains of mice (outbred, inbred, transgenic, mutants), 4 strains of rats and 1 stock of hamsters, on spontaneous tumors (i.e., non-exposed to any carcinogenic agent) or tumors induced by 16 chemical carcinogens of different classes (e.g., polycyclic aromatic hydrocarbons, nitroso compounds, estrogens, etc.) and of direct or indirect action (i.e., needing metabolic transformation to become proximal carcinogens) and induced by total body X-rays and γ-irradiation, viruses, bacteria, genetic modifications or special high-fat diet, these carcinogens being introduced using one-stage or two-stage protocols of carcinogenesis. Biguanides were administered via 5 various routs - oral gavage, subcutaneous or intraperitoneal injections, with drinking water or diet – in various doses and regimens [9, 10].

In the majority of the cases (86%) the administration of antidiabetic biguanides suppressed carcinogenesis (decreased the incidence and number or size of tumors, inhibited their metastatic potential, increased tumor latency or the rate of differentiation), whereas in 14% of cases the inhibitory effect of biguanides was not observed. It is worthy to note that there were no cases of stimulatory effect of antidiabetic biguanides on carcinogenesis in rodentds [9, 10]. There are hundreds of reviews and meta-analyses of results of clinical trials on the effect of antidiabetic biguanides on the risk of cancer in humans. It was concluded that they had no beneficial effect in all types of cancers, and further studies were needed in this field [11, 12]. These data allow to conclude that there is sufficient evidence of the anticarcinogenic potential of these drugs in rodents, and limited evidence of the anti-cancer effect of biguanides in humans. Have 50 years been enough to prove the brilliant Dilman’s idea?
